# Twenty years on from the introduction of the high risk strategy for stroke and cardiovascular disease prevention: a systematic scoping review

**DOI:** 10.1111/ene.16157

**Published:** 2023-11-27

**Authors:** Valery L. Feigin, Sheila C. Martins, Michael Brainin, Bo Norrving, Saltanat Kamenova, Azhar Giniyat, Aida Kondybayeva, Daulet K. Aldyngurov, Magripa Bapayeva, Murat Zhanuzakov, Graeme J. Hankey

**Affiliations:** ^1^ National Institute for Stroke and Applied Neurosciences, School of Clinical Sciences Auckland University of Technology Auckland New Zealand; ^2^ Institute for Health Metrics Evaluation University of Washington Seattle Washington USA; ^3^ Hospital de Clínicas de Porto Alegre Hospital Moinhos de Vento Porto Alegre Brazil; ^4^ Department of Neuroscience and Preventive Medicine Danube University Krems Krems Austria; ^5^ Department of Clinical Sciences Skåne University Hospital, Lund University Lund Sweden; ^6^ Department of Neurology Skåne University Hospital, Lund University Lund Sweden; ^7^ Asfendiyarov Kazakh National Medical University Almaty Republic of Kazakhstan; ^8^ Minister of Healthcare of the Republic of Kazakhstan Astana Republic of Kazakhstan; ^9^ Department of Science and Human Resource Ministry of Healthcare of the Republic of Kazakhstan Astana Republic of Kazakhstan; ^10^ Department of Internal Medicine Kazakhstan Medical University «KSPH» Almaty Republic of Kazakhstan; ^11^ Higher School of Medicine al‐Farabi Kazakh National University Almaty Republic of Kazakhstan; ^12^ Perron Institute Chair in Stroke Research, Medical School University of Western Australia Perth Western Australia Australia; ^13^ Perron Institute for Neurological and Translational Science Perth Western Australia Australia

**Keywords:** cardiovascular disease, high risk strategy, prevention, stroke, trials

## Abstract

**Background and purpose:**

Early this century, the high risk strategy of primary stroke and cardiovascular disease (CVD) prevention for individuals shifted away from identifying (and treating, as appropriate) all at‐risk individuals towards identifying and treating individuals who exceed arbitrary thresholds of absolute CVD risk. The public health impact of this strategy is uncertain.

**Methods:**

In our systematic scoping review, the electronic databases (Scopus, MEDLINE, Embase, Google Scholar, Cochrane Library) were searched to identify and appraise publications related to primary CVD/stroke prevention strategies and their effectiveness published in any language from January 1990 to August 2023.

**Results:**

No published randomized controlled trial was found on the effectiveness of the high CVD risk strategy for primary stroke/CVD prevention. Targeting high CVD risk individuals excludes a large proportion of the population from effective blood‐pressure‐lowering and lipid‐lowering treatment and effective CVD prevention. There is also evidence that blood pressure lowering and lipid lowering are beneficial irrespective of blood pressure and cholesterol levels and irrespective of absolute CVD risk and that risk‐stratified pharmacological management of blood pressure and lipids to only high CVD risk individuals leads to significant underuse of blood‐pressure‐lowering and lipid‐lowering medications in individuals otherwise eligible for such treatment.

**Conclusions:**

Primary stroke and CVD prevention needs to be done in all individuals with increased risk of CVD/stroke. Pharmacological management of blood pressure and blood cholesterol should not be solely based on the high CVD risk treatment thresholds. International guidelines and global strategies for primary CVD/stroke prevention need to be revised.

## INTRODUCTION

Evidence from the Global Burden of Disease studies suggests that prevalent cases of total cardiovascular disease (CVD) (including stroke) nearly doubled from 271 million in 1990 (95% uncertainty interval 257–285 million) to 523 million in 2019 (95% uncertainty interval 497–550 million) [1]. Despite a consistent decline in age‐standardized CVD (including stroke) mortality rates globally in the last half of the 20th century [1], there has been a subsequent deceleration in the decline, and now an overall flattening of the decline in the past 5 years [2]. Since 2010, age‐standardized CVD mortality rates have even increased in many locations (e.g., United States, Mexico, UK) [1, 3], and there has been a significant increase in the age‐standardized incidence of stroke in young individuals (under 55) in high‐income countries [4, 5]. Globally, the age‐standardized prevalence of high systolic blood pressure (SBP) [1], age‐standardized disability‐adjusted life‐years lost due to high fasting plasma glucose [1], high body mass index [1] and age‐standardized incidence and prevalence of diabetes mellitus are also increasing [6]. These data strongly suggest inadequate risk prediction and risk factor control in the population, and particularly the younger population, that demands innovative public health solutions [7].

Some 40 years ago, the strengths and weaknesses of population‐wide and high risk strategies of CVD prevention were thoroughly delineated by Rose [8]. The population‐wide approach involved identifying (and treating, as appropriate) all at‐risk individuals, whereas the high risk approach involved identifying and treating individuals who exceeded arbitrary thresholds of predicted absolute risk of CVD. The high CVD risk approach is valid and suitable for selecting people at high risk of acute CVD and monitoring their progress in CVD prevention, but whether it is also effective in preventing CVD remains unknown. Rose considered both approaches to be complementary, rather than mutually exclusive, emphasizing that the fundamental limitation of the high risk CVD prevention strategy was that it misses a large number of preventable CVD cases [8]. Concurrently, there were—and remain—public health experts and senior epidemiologists opposing this strategy [9, 10, 11, 12, 13, 14]. As Capewell highlighted in 2008, the greatest harm arising from high risk strategies is misleading professionals, planners and politicians into thinking that by implementing the high risk strategy of CVD prevention in practice they can tick the ‘mission accomplished’ box for preventing CVD [9]. Capewell argued that screening for high risk individuals represents a costly and relatively ineffective strategy that distracts from cheaper and more effective population‐based policy interventions which benefit the entire population [9].

Despite these concerns, the high risk strategy for CVD prevention was soon recommended in several national [15, 16, 17] and international guidelines [18] whereby quantitative treatment thresholds, based on an individual's predicted absolute CVD risk, were the key determinants of the indication for pharmacological treatment of raised blood pressure and raised blood cholesterol. Since 2000, wider use of the high risk strategy of CVD prevention was advocated and, in 2005, there was a call to use absolute CVD risk prediction score thresholds routinely as the sole criteria for prescribing blood‐pressure‐lowering and blood‐lipid‐lowering pharmacological treatment [17]. Although supported by only observational and modelling studies, this high CVD risk strategy was quickly adopted by major international [19, 20, 21, 22, 23, 24] and several national primary CVD prevention guidelines [25, 26, 27, 28], including the World Health Organization (WHO) [29].

Twenty years from the introduction of the high risk strategy for stroke and CVD prevention into practice seems a sufficient time period to identify and map the available evidence accumulated to date, for and against the shift in the focus of primary stroke and CVD prevention away from populations and towards individuals at high risk. Specifically, in our systematic scoping review an attempt is made to identify and appraise publications related to uptake, cost‐effectiveness, medical effectiveness, risk stratification strategies and treatment thresholds of the high CVD risk strategy for primary stroke/CVD prevention.

## METHODS

**FIGURE 1:**
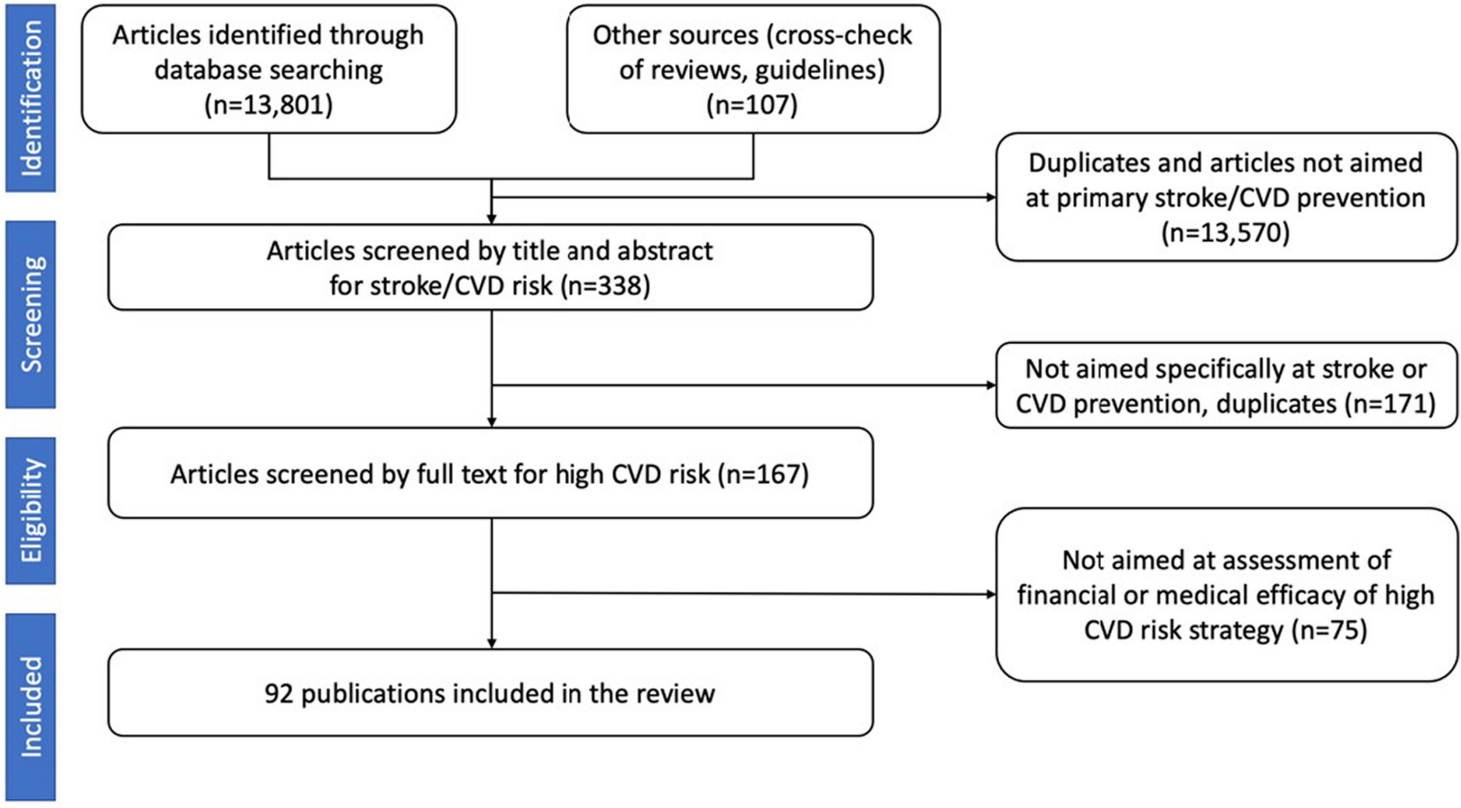
PRISMA flowchart summarizing the literature screening process.

Established guidelines for conducting a systematic scoping review were followed [30]. Our literature search of Scopus, MEDLINE, Embase, Google Scholar and the Cochrane Library databases to identify relevant articles published in any language from January 1990 to August 2023 used the following search terms in title, abstract or keywords: ‘cardiovascular disease’, ‘stroke’, ‘transient ischaemic attack (TIA)’, ‘cerebrovascular’ or ‘CVD’ AND ‘primary prevention’, ‘high (CVD) risk’, ‘efficacy’, ‘effectiveness’, ‘efficiency’, ‘randomis(z)ed controlled trial (RCTs)’, ‘cost’, ‘clinical trial’, ‘risk threshold(s)’, ‘stratification’, ‘screening’, ‘epidemiology’, ‘cohort studies’, ‘trend(s)’, ‘population‐based’ or ‘burden’. The reference list of identified pivotal articles was manually searched, and additional relevant publications were retrieved for analysis. In selecting epidemiological studies preference was given to the Global Burden of Disease and population‐based studies. PRISMA guidelines [31] were followed for reporting the literature search process (Figure 1). Of the total 13,789 publications initially identified, only 92 studies fulfilled the inclusion criteria and were included in the review.

## RESULTS

### Cost‐effectiveness and uptake of the high CVD risk screening

Although it has long been argued that the benefits of population CVD screening must be established through properly conducted trials [32], to date no RCTs evaluating the cost‐effectiveness of high CVD risk screening have been found. The results of health economic modelling studies over the last 20 years have been conflicting. Whilst some microsimulation studies showed that universal screening for CVD risk and treatment of people at high CVD risk are not the most effective options for primary prevention of CVD overall compared to population‐wide strategies [10, 33], another modelling study suggested that, with 100% detection (including previously undiagnosed diabetes) and effective treatment of all individuals with high CVD risk, it would be possible to save £61 billion and prevent 5.2 million CVD cases over 25 years [34]. Results of another modelling study suggested that, even if high CVD risk screening systems were effective, and 100% of individuals in the population with a 10‐year CVD risk of ≥30% (6% of the population) were identified, and all of these individuals were appropriately treated, the incidence of major CVD would be reduced by, at most, 11% [35].

However, in practice the uptake of high CVD risk screening programmes is low (about 17%–20%) even in high‐income countries (e.g., England, Australia, New Zealand) where such programmes are virtually mandatory for health practitioners [36, 37], and their implementation in practice is expensive (e.g., the total cost of the basic Health Check of 1.5 million people using QRISK2 in the UK costs almost £31 million) [38]. For example, in Australia, even after the introduction in 2019 of an $85.60 incentive for medical practitioners for a dedicated CVD risk consultation that lasts at least 20 min, the total number of assessments for high CVD risk did not change [36]. In addition, high CVD risk screening programmes require considerable efforts and cost from society [14] (and, in most countries, from individuals) and are therefore unlikely to be widely implemented in countries with limited resources. For example, in England in 2015 the National Health Service (NHS) Health Checks cost taxpayers £450 million a year [13] but could prevent only one additional fatal CVD event every year for every 4762 (95% confidence interval [CI] 4167–5263) people who attend a Health Check [39], raising a concern about the cost and cost‐effectiveness of the intervention [40]. A cost‐neutral effect of incentivizing CVD prevention by payment for CVD risk calculation, assessment and reduction was recently shown in the Million Hearts Model trial in the USA [41].

Although non‐laboratory CVD risk screening tools such as the non‐laboratory INTERHEART risk score [42] and WHO CVD risk charts [43] can be introduced by non‐physician health workers, their uptake and effectiveness in about half of the world's poorest populations living on less than US$7.00 per person per day [44] is likely to be very limited.

### Medical effectiveness of the high CVD risk strategies

Whilst the WHO CVD risk charts [43] for risk‐based CVD management [45] could be used where feasible for targeting individuals with high CVD risk, this strategy alone leaves the majority of people at risk neglected (over 80% of all CVDs/stroke events occur in these low CVD risk people) [46, 47, 48, 49]. Although it was postulated that the benefits of CVD risk‐reducing interventions are proportional to the estimated CVD risk [25], no robust evidence has been found for medical effectiveness of targeted high CVD risk screening compared to opportunistic/conventional screening for CVD risk. Although the Million Hearts Model cluster RCT in the USA [41], which encouraged and paid for CVD risk assessment and reduction, showed reduction in first‐time strokes and myocardial infarctions, the study did not directly test efficacy of the high CVD risk prevention strategy, did not show effects on the primary outcome of CVD events or the outcome of CVD events or CVD deaths amongst high risk beneficiaries alone, and suffered from a number of significant limitations [50]. On top of the limitations already acknowledged by the authors, the internal validity of the trials was not high as a third of randomized clusters did not provide CVD risk estimates, and only about half of beneficiaries in the intervention group used the CVD screening tool. Moreover, participating organizations volunteered for random assignment into the model, although it is well known that organizations/participants who enter such trials are likely to be motivated to succeed with or without incentive, generally biasing results toward the null. Finally, medication adherence was not assessed and changes in the prevalence of risk factors were not reported; therefore it remains unclear why CVD incidence and mortality decreased in the incentivized group but not in the control group. A recent WHO systematic review of 14 large high‐quality RCTs for reducing the burden of CVDs (including stroke) clearly showed that population‐level screening programmes for CVD risk and CVD risk factors have had no impact on lowering CVD morbidity and mortality in the general population [51].

The Inter99 RCT (59,616 people aged 30–60 years followed for 10 years) [52] was specifically designed to determine the effects of screening for CVD risk and risk factors and lifestyle counselling on incidence of ischaemic heart disease (IHD) in the general population. It found no significant difference between the intervention and control groups in the risk of IHD (hazard ratio [HR] 1.03, 95% CI 0.94−1.13), stroke (HR 0.98; 95% CI 0.87−1.11), combined IHD and stroke (HR 1.01; 95% CI 0.93−1.09) and total mortality (HR 1.0; 95% CI 0.91−1.09). However, the trial involved computer simulation techniques to reach a substantial level of CVD risk of the study participants and a counselling intervention was not supported by additional drug treatment; therefore the study results should be interpreted with caution. A subsequent Cochrane meta‐analysis [53] of 15 RCTs comparing the effect of health checks (screening for more than one disease or risk factors) with no health checks in a total of 251,891 adults found there were no beneficial effects of general health checks over 1–15 years’ follow‐up for total mortality (risk ratio [RR] 1.00, 95% CI 0.97−1.03; *I* ^2^ = 0%), CVD mortality (RR 1.05, 95% CI 0.94−1.16; *I* ^2^ = 65%), ischaemic heart disease incidence (RR 0.98, 95% CI 0.94−1.03; *I* ^2^ = 11%) or stroke incidence (RR 1.05, 95% CI 0.95−1.17; *I* ^2^ = 53%). However, the review was mainly based on the old, heterogeneous studies that were prone to bias and was focused on general health checks and not specifically on detection of high CVD risk individuals.

### Risk stratification management and treatment thresholds

One of the major objectives of high CVD risk screening is to identify individuals for blood‐pressure‐lowering and lipid‐lowering pharmacological treatment based on an arbitrarily determined absolute CVD risk threshold [54]. Before the introduction in 2021 of the WHO guidelines [55] for pharmacological management of elevated blood pressure, hypertension management was solely based on the CVD risk threshold (e.g., 5‐year absolute CVD risk ≥15%) [25]. Therefore, people with hypertension (SBP ≥140 mmHg and/or diastolic blood pressure ≥90 mmHg) who had a 5‐year absolute CVD risk <15% were exempted from receiving blood‐lowering medication. However, elevated blood pressure is the single most important cause of stroke/CVD burden worldwide [56], and isolated systolic hypertension is observed in 32% of healthy adults [57], is highly prevalent in the elderly and is a major cause of mortality and morbidity [58]. According to the PREDICT CVD risk calculator [59], people aged 60–80 years old with isolated SBP ranging from 140 to 184 mmHg and no additional risk factors have a 5‐year CVD risk <15%. Implementation of the high risk strategy would leave these people without effective blood‐pressure‐lowering treatment.

In addition, categorizing people into low and moderate CVD risk may give them a false reassurance that they are protected from stroke/CVD, thus attenuating any motivation to control their risk factors [60]. There is also evidence that existing CVD risk score estimates perform poorly in the developing countries and may lead to misclassification of individuals who do and do not require treatments [61, 62]. For example, a 45‐year‐old man who smokes cigarettes, is physically inactive and has usual blood pressure of 140/90 mmHg has a 10‐year absolute CVD risk of 2.4%–2.5% (according to the ACC/AHA ASCVD and QRISK®2‐2014 algorithms) and a 5‐year absolute CVD risk of 1.3% (according to the PREDICT algorithm). This individual is categorized as having a low risk of CVD and, according to the existing absolute CVD risk guidelines [63], would not be offered a pharmacological treatment for hypertension.

Although initial justification for the high risk strategy was that applying absolute CVD risk treatment thresholds (considered a combined effect of various risk factors) would allow more individuals with raised blood pressure and raised blood cholesterol to receive blood‐pressure‐ and lipid‐lowering treatment than treatment based just on the raised level of these risk factors [54], the evidence to date showed the opposite. Indeed, in a recent (2022) large (*n* = 3,337,314) cross‐sectional study of 45–74‐year‐old adults in Australia [64], 41% of individuals with low CVD risk and blood pressure above 140/90 mmHg were not managed with antihypertensive medications. Yet, this group of people, aged 45–74 with low to moderate CVD risk, constitutes >80% of the population [25, 46, 47, 48, 49, 65, 66] and accounts for a substantial proportion (up to 90%) of CVD events [8, 67, 68]. Screening for those at high CVD risk and targeting risk‐stratified pharmacological management of blood pressure and lipids to only high CVD risk individuals is also likely to exacerbate socio‐economic inequalities in CVD prevention [10]. A significant underuse of statins for lipid‐lowering therapy in Europe [69] due to the introduction of the updated SCORE2 10‐year CVD risk prediction algorithm [70] has called for revisiting 10‐year risk to guide statin therapy or even stopping use of 10‐year predicted CVD risk as the main starting point for statin recommendations for improving identification of people eligible for statins in primary CVD prevention [71].

Previous meta‐analyses of RCTs (2012 and 2014) provided evidence that the absolute benefits of blood‐pressure‐ and lipid‐lowering drugs are largely determined by patient's predicted pre‐treatment vascular risk [72, 73]. However, a more recent meta‐analysis (2021) of individual participants' data (*n* = 358,707) from 51 randomized clinical trials [74] convincingly showed a beneficial effect of pharmacological blood‐pressure‐lowering treatment across a wide range of ages with no evidence to suggest that the relative risk reductions for prevention of major CVD events (including stroke) vary by baseline systolic or diastolic blood pressure levels, down to less than 120/70 mmHg. A strong beneficial effect of blood‐pressure‐ and lipid‐lowering treatment regardless of the baseline level of blood pressure or lipids was also demonstrated in the large (12,705 study participants with intermediate CVD risk) multicentre HOPE‐3 trial [75] and subsequent meta‐analysis of this and two other large RCTs (TIPS‐3 [76] and PolyIran [77]), with a total of 18,162 participants [78]. Evidence from these studies supports lowering blood pressure and cholesterol for primary CVD prevention, irrespective of blood pressure and cholesterol levels and irrespective of the level of absolute CVD risk [79].

## DISCUSSION

Our scoping review shows that evidence to support the high CVD risk prevention strategy remains lacking and does not meet WHO criteria for resource‐effective CVD prevention strategies to be implemented in practice, namely (1) a substantial effect on health, (2) strong evidence for cost‐effectiveness, (3) feasibility of implementation in settings with different levels of resources and (4) political and financial feasibility for scale‐up [11, 80]. However, despite the uncertainties and lack of robust evidence of the efficacy of the high risk strategy it has continued to prevail and large resources and efforts are spent on the implementation of this strategy, improving accuracy of the prediction algorithm, re‐calibrating it for different populations and refining (actually reducing) the treatment thresholds for blood‐pressure‐ and lipid‐lowering pharmacological treatments. It is believed that all these activities are only justified if there is robust evidence of the effectiveness of the high CVD risk prevention strategy in the first place. This is the fundamental principal of evidence‐based medicine.

Whilst the WHO CVD risk charts [43] for risk‐based CVD management [45] could be used where feasible for targeting individuals with high CVD risk and applying blood‐pressure‐ and lipid‐lowering treatment thresholds for primary prevention for an identified minority, this strategy alone leaves the majority of people at risk neglected, as 80% of all CVDs/stroke events occur in these people [46, 47, 48, 49]. High CVD screening does squeeze primary prevention resources and bring false reassurance to low and moderate CVD risk individuals, which is opposite to early warning and timely preventative intervention.

**FIGURE 2:**
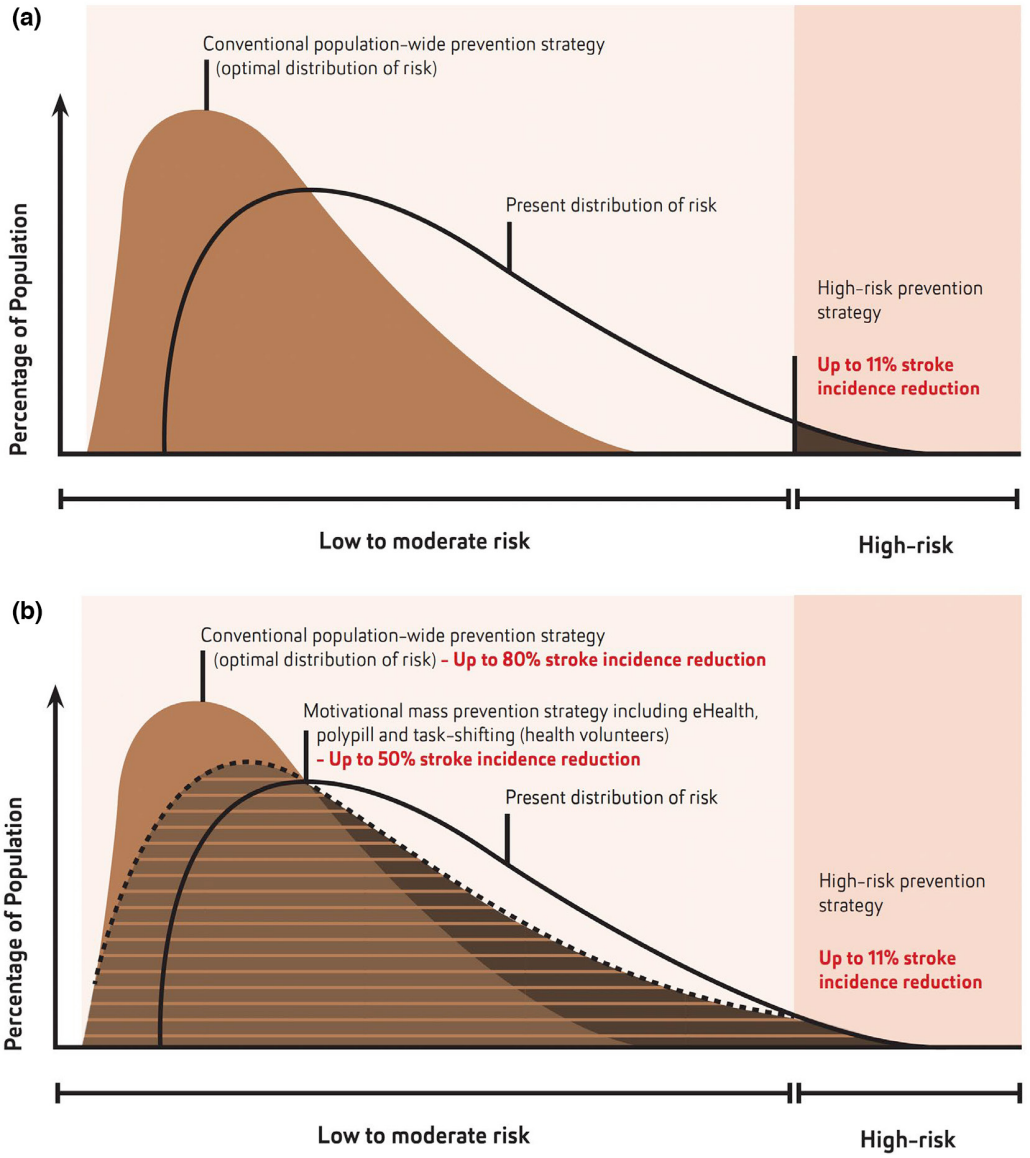
Optimal shift in the distribution of stroke and cardiovascular disease (CVD) risks that could be achieved with the adaptation of the recommended changes in stroke/CVD prevention strategies from high CVD risk prevention strategy to motivational mass prevention strategies in all individuals with elevated risk of stroke/CVD, regardless of the level of risk (e.g., eHealth, polypill and task‐shifting in stroke/CVD prevention/screening to health workers/volunteers) and wider implementation of population‐wide prevention strategies. (Modified from Feigin et al. [97] and Owolabi et al. [56], with permission.) Areas shaded in brown show a theoretically possible proportion of the population that could benefit from (a) high CVD risk prevention strategy (at best 11% stroke/CVD risk reduction) and (b) a motivational mass individual risk prevention strategy regardless of the CVD risk level (e.g., use of mobile applications to reduce lifestyle and other risks, polypill and task‐shifting), additional 40% reduction in stroke/CVD incidence and population‐wide prevention strategies, plus 30% stroke/CVD incidence reduction in addition to the motivational mass individual risk prevention strategy regardless of the level of increased CVD risk.

To be effective, stroke/CVD prevention should be focused on population‐wide strategies (such as addressing poverty, socio‐economic inequalities and reducing exposure to various environmental, metabolic and lifestyle risk factors in the whole population). Such strategies should be enhanced by culturally appropriate motivational strategies on the individual level to include all people at increased level of CVD risk [11] using simple to use, validated screening tools for community people and health professionals that would not only allow estimation of the risk but also provide evidence‐based person‐centred preventative recommendations and motivate people to control their risk factors [81, 82, 83, 84]. Assessment of the effectiveness of such strategies should be based on changes in the incidence of stroke/CVD in the population [85]. Providing estimates of the lifetime risk and years lived free of total CVD [86, 87] as a motivational tool to control risk factors in individuals with low and medium CVD may also be helpful. In addition, task shifting in primary stroke/CVD prevention to health workers/volunteers or nurses (nurse educators), and use of affordable polypill containing fixed doses of blood‐pressure‐lowering and blood‐lipid‐lowering medications with or without aspirin in adults at intermediate CVD risk are also recommended (Figure 2) [88]. Should these evidence‐based preventive stroke/CVD strategies be freely accessible and supported by universal health coverage [89], this could allow about a 40%–70% increase in the efficiency of stroke reduction on the individual and population level, respectively [90, 91, 92, 93, 94], compared to a high CVD risk prevention strategy [56]. Governments should allocate a fixed proportion of annual health funding for prevention of stroke/CVD, which could come from taxation on tobacco, salt, sugar and alcohol [95].

**FIGURE 3:**
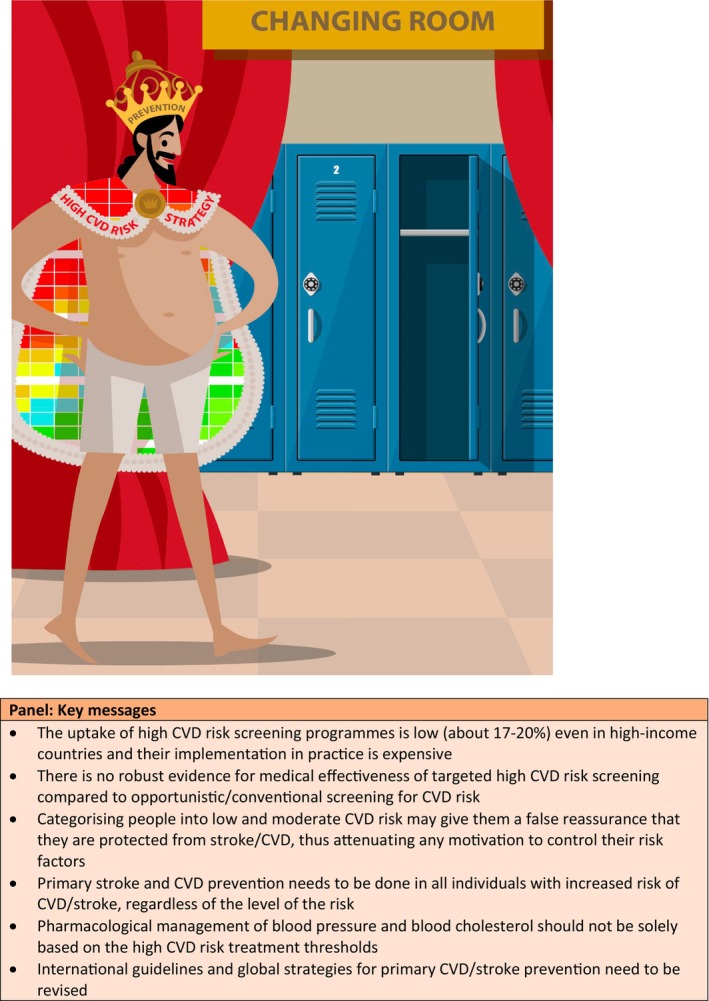
Time to dress the Emperor of Medicine—Prevention.

Our key messages are presented in the panel. Evidence to date strongly suggests that pharmacological management of blood pressure and blood cholesterol should not be based solely on high CVD risk treatment thresholds, and systematic population‐level screening programmes for CVD risk and CVD risk factors are not medically effective and cost‐effective [51]. As high risk CVD management strategies were initially endorsed by the WHO Member States as the mainstream strategy for primary CVD (including stroke) prevention on the individual level, only WHO Member States may alter/modify these strategies. Therefore, the WHO Member States are being called to initiate the process. The first step in responding to the accumulated evidence about high risk management treatment thresholds has recently been made by the WHO with the publication of new guidelines for the pharmacological treatment of hypertension in adults [55] in which criteria for blood‐pressure‐lowering treatment are not solely based on the absolute CVD risk threshold. Recent (2023) inclusion of polypill for stroke/CVD prevention in the WHO list of essential medicines is another important step in the right direction [96]. It is now time to complete the proper ‘evidence‐based’ dressing of the Emperor of Medicine—Prevention and revise national and international guidelines and global strategies for stroke/CVD prevention on the individual and population levels (Figure 3).

## AUTHOR CONTRIBUTIONS

**Valery L. Feigin:** Conceptualization; writing – original draft; writing – review and editing; methodology; formal analysis; resources. **Sheila C. Martins:** Conceptualization; writing – review and editing; conceptualization; methodology. **Michael Brainin:** Conceptualization; writing – review and editing. **Bo Norrving:** Conceptualization; writing – review and editing. **Saltanat Kamenova:** Conceptualization; writing – review and editing. **Azhar Giniyat:** Conceptualization; writing – review and editing. **Aida Kondybayeva:** Conceptualization; writing – review and editing. **Daulet K. Aldyngurov:** Conceptualization; writing – review and editing. **Magripa Bapayeva:** Conceptualization; writing – review and editing. **Murat Zhanuzakov:** Conceptualization; writing – review and editing. **Graeme J. Hankey:** Conceptualization; methodology; formal analysis; resources; writing – review and editing. **Sheila C. Martins:** Conceptualization; writing – review and editing; conceptualization; methodology.

## FUNDING INFORMATION

None.

## CONFLICT OF INTEREST STATEMENT

The authors declare no competing interests.

## Data Availability

The data that support the findings of this study are available from the corresponding author upon reasonable request.
